# Mangosteen Seed Fat: A Typical 1,3-Distearoyl-Sn-2-Linoleoyl-Glycerol-Rich Fat and Its Effects on Delaying Chocolate Fat Bloom

**DOI:** 10.3390/foods14040557

**Published:** 2025-02-07

**Authors:** Xueying Hou, Yuhang Chen, Lai Wei, Jun Jin

**Affiliations:** State Key Laboratory of Food Science and Resources, School of Food Science and Technology, Jiangnan University, Wuxi 214122, China

**Keywords:** mangosteen seed fat, fat bloom, StLSt, compatibility, cocoa butter

## Abstract

Mangosteen seed fat (MSF), a novel tropical seed fat, predominantly comprises 1,3-distearoyl-2-linoleoyl-glycerol (StLSt) and 1,3-distearoyl-2-oleoyl-glycerol (StOSt). The fat was blended with cocoa butter (CB) in proportions of 5%, 25% and 60% in the present study, and the binary blends achieved acceptable miscibility. It was indicated that StLSt could be mixed well with the symmetrical monounsaturated triacylglycerols in CB, especially StOSt, 1-palmitoyl-2-oleoyl-3-stearoyl-glycerol (POSt) and 1,3-dipalmitoyl-2-oleoyl-glycerol (POP). Although the solid fat contents of the binary blends gradually decreased with the addition of MSF, which resulted from low-melting triacylglycerols in MSF, the well-compatible fat matrix contributed to keeping their desirable melting behaviors and hardness at hot temperatures. A chocolate fat bloom test showed that replacing CB with 25–60% MSF improved fat-bloom-resistant stabilities effectively. The effective steric hindrance of StLSt crystals may improve fat compatibilities and further delay liquid–oil migration and recrystallization in chocolates during temperature fluctuations.

## 1. Introduction

Cocoa butter (CB) predominantly comprises 1,3-dipalmitoyl-2-oleoyl-glycerol (POP; 17.5–22.6%), 1-palmitoyl-2-oleoyl-3-stearoyl-glycerol (POSt; 35.8–41.4%) and 1,3-distearoyl-2-oleoyl-glycerol (StOSt; 22.8–31.1%) [1]. CB can form six crystal forms, among which the β_2_-V crystal is typically obtained through a precise tempering process. Ghazani and Marangoni [2] discovered that the conversion of β_2_-V to β_1_-VI in CB is related to POSt, which might be closely associated with fat bloom.

The price of cocoa has increased significantly due to environmental, political and economic factors, and the substitution of CB in chocolate has once again become a hot topic in the food industry [3,4]. Consequently, the most crucial aspect to address is the chocolate bloom defect. Koizumi et al. [5,6] found that controlling storage temperature could regulate crystallization behaviors of CB, thereby inhibiting fat bloom in cocoa butter substitute-based compound chocolates, but the constant temperature increased the cost of the product. Cocoa butter alternatives, mainly cocoa butter equivalents (CBEs) and cocoa butter improvers (CBIs), possess similar triacylglycerol types to CB, significantly enhancing their compatibilities, and are suggested to make premium chocolates together with CB. In particular, CBI has a high proportion of StOSt and is generally utilized to improve the hardness of chocolates, therefore contributing to improving fat bloom stabilities [7]. Jin et al. [8] increased the melting point of chocolates by adding mango kernel fat-based fats (riched in StOSt) to solve the fat bloom problem. Fukami et al. [9] used palm-based replacers to promote the formation of molecular compounds in the system and delay the transformation of β′ into the β crystal form, thus delaying fat bloom. Many fat alternatives are obtained from tropical and subtropical xylophyta. Besides traditional CBEs and CBIs, some specialty fats have been developed. Mangosteen (*Garcinia mangostana* L.) is a tropical evergreen tree belonging to the family *Clusiaceae* [10]. Mangosteen has smooth and round fruits that turn dark purple or red-purple when mature. The flesh, rind and seed account for 25–29%, 60–65% and 6–11%, respectively. The mangosteen peel contains various secondary metabolites, which exhibit excellent properties in antioxidation and anti-tumor activities [11]. Nevertheless, mangosteen seeds are often discarded or underutilized [12]. In recent years, mangosteen seed fat (MSF) has started to attract attention [13,14,15]. Chen et al. [15] further reported that 1,3-distearoyl-sn-2-linoleoyl-glycerol (StLSt), StOSt and 1-palmitoyl-2-linoleoyl-3-stearoylrac-glycerol (PLSt) account for 31.67%, 31.47% and 12.31% of MSF, respectively. The fat shows acceptable compatibility with cocoa butter. In addition, MSF is derived from plants, and adding it to chocolate will not affect its consumption for vegans. However, whether MSF can play a role similar to CBIs in fat bloom remains to be explored.

Another notable point is that, as one of the main triglyceride components in MSF, StLSt differs from StOSt only in that the sn-2 position is linoleic acid. Takeuchi et al. [16,17] reported that StLSt presents four crystal forms: sub-α₂, sub-α₁, α and γ. Among them, only the γ crystal form is a triple-chain-length structure and has similar structural characteristics to StOSt (Appendix A Table A1). Further exploration revealed that when the concentration of StLSt exceeds 30%, it can restrict the separation of StOSt from the solid solution phase, thereby stabilizing the fat system. It is hypothesized that this is dominated by the olefinic interactions between oleoyl and linoleoyl chains. From the perspective of restricting crystal form transformation, the crystallization characteristics of StLSt have considerable advantages in improving chocolate bloom-resistant abilities.

The present study focuses on analyzing fatty acid compositions and triglyceride species of MSF using chromatographic techniques. The effects of MSF on delaying chocolate bloom are explored with storage tests, and possible reasons were further analyzed from the perspectives of melting point and hardness of chocolates, as well as melting behaviors, fat compatibilities and crystal morphologies of fat matrix.

## 2. Materials and Methods

### 2.1. Materials

CB was purchased from Shaoxing Qili Xingguang Cocoa Products Co., Ltd. (Shaoxing, China). Mangosteen (*Garcinia mangostana* L.) was bought from the local supermarket (Wuxi, China). Fresh mangosteen seeds were vacuum-dried at 50 °C until the weight remained constant, and the dry weight W_0_ was recorded. The fully ground seeds were mixed with hexane at a material-to-solvent ratio of 1:5 and extracted four times at 55 °C for 1 h each time. The hexane was removed using a rotary evaporator under a vacuum at 80 rpm and 50 °C, and the weight of the fat W_1_ was recorded. The value of “W_1_/W_0_”, 47.11%, was taken as the fat content of the mangosteen seeds. Due to its high fat content, both solvent extraction and direct pressing were acceptable in the industry. The fat was mixed with CB in proportions of 5%, 25% and 60, i.e., named M1, M2 and M3, respectively.

### 2.2. Determination of Fat Acid Composition

The fatty acid composition was analyzed based on the previous method [18]. Fat (50 mg) was dissolved in 2 mL hexane and mixed with potassium hydroxide solution (1 mL, 2 mol/L). Then, samples were centrifuged at 10,000 rpm, the supernatant was collected and anhydrous sodium sulfate was added to remove water. The detection conditions were as follows: carrier gas (nitrogen), 1 mL/min; split ratio, 1:100; injector temperature, 250 °C; detector temperature, 260 °C; column temperature, 80 °C for 0.5 min, 80 °C to 165 °C at 40 °C/min, holding at 165 °C for 1 min, 165 °C to 230 °C at 2 °C/min, holding at 230 °C for 2 min. The samples were detected by gas chromatography (7820 A, Agilent, Santa Clara, CA, USA), equipped with a flame ionization detector (FID) and a DB-Fast FAME capillary column (0.25 µm, 0.25 mm × 30 m, Agilent, USA). The composition of fatty acids was characterized by 37-component FAME mixed standards (Sigma, St. Louis, MO, USA) and quantified using the area normalization method.

### 2.3. Analysis of Triacylglycerol Species

The samples (6 mg) were dissolved in trichloromethane (2 mL) and filtered through a nylon membrane (0.22 µm). Triacylglycerols were detected by a Waters 1525 liquid chromatographic system (Waters Corp., Milford, MA, USA), equipped with an evaporative light-scattering detector and a Develosil C30 column (250 mm × 4.6 mm, Nomura chemical, Seto, Aichi, Japan). The solvents were acetone, acetonitrile and isopropanol, respectively, and samples were eluted with a volume ratio of 5:3:2 at 1.2 mL/min. The detector temperature was set to 55 °C at a gain of 1, and the carrier gas flow rate was set to 1.8 mL/min [19]. The triacylglycerols were qualitatively determined by analyzing the peak of common oils (soybean oil, cocoa butter, etc.).

### 2.4. Determination of Solid Fat Content

In order to determine the crystalline state of chocolates, further studies were carried out corresponding to all the fats used in chocolate. The samples (2 g) were placed in NMR tubes and stored at 80 °C for 0.5 h to eliminate crystal history. Melted samples were treated according to the tempering procedure in “2.6 preparation of chocolates” and then stored at 20 °C for 1 day. SFC values of the binary fats were determined [15] using a minispec SFC analyzer (NE 3759, Bruker, Karlsruhe, Germany). The samples were stabilized at 0 °C for 1.5 h and measured from 0°C to 40°C with 5 °C (0.5 h) increments each time.

In such symmetrical triacylglycerol systems, the difference between the actual SFC (SFCa) and the theoretical SFC (SFCt), known as ΔSFC, may provide the performant estimation compossibilities of the binary fats with the following equation [20]:

ΔSFC = SFCa − SFCt

SFCt = xSFCx + ySFCy

where SFCx and SFCy are the actual SFC values of cocoa butter and MSF at the corresponding temperature, and x and y are the mass fractions of the two components in the mixture.

### 2.5. Determination of Crystal Morphology

Chocolate fats were melted at 80 °C for 0.5 h and then placed onto preheated slides by capillary tubes. Following the chocolate tempering procedure, the samples were transferred to the corresponding temperature incubator in turn and stored at 20 °C after tempering. The images were obtained by a polarized light microscope (PL-180, Shangguang, Shanghai, China) with a 20- or 40-time objective according to the crystal diameter. Four parallel samples were prepared for each fat, and the representative crystal morphologies were selected for analysis.

### 2.6. Preparation of Chocolates

The prepared fats (CB, M1, M2 or M3; 12.9%), 46.9% cocoa liquor, 39.5% powdered sugar and 0.7% soy lecithin, were used as raw materials [8]. The chocolate ingredients were thoroughly mixed and fully ground with a mortar at 60 °C for 6 h to ensure that the sugar particles were fine enough. Tempering was performed on a chocolate tempering machine (Revolation 2B, Chocovision, New York, NY, USA), and the typical procedures were as follows: the liquid chocolate was cooled to 20 °C and kept for 5 min. It was then heated to 42.2 °C in 1 min, cooled to 32.2 °C in 4 min and further cooled to 28.0 °C in 3 min. Finally, the temperature was raised to 31.5 °C within 5 min. The well-tempered material was poured into a square mold and demolded after 24 h at 20 °C.

### 2.7. Chocolate Bloom Test During Storage

The chocolates were stored at 17 °C and 32 °C in 12 h cycles for 60 days [8]. Each type of chocolate was set to three parallel groups, and their states were recorded during low-temperature storage. The surface morphologies of the chocolates were photographed daily under a fixed light source. L, a and b values of the chocolate surfaces were measured by a colorimeter (NR110, Shenzhen ThreeNH Technology Co., Ltd., Guangzhou, China), and the whiteness indices (WIs) were calculated by the following formula:

$$\mathrm{WI}=100-\sqrt{{\left(100-L\right)}^{2}+a^{2}+b^{2}}$$

### 2.8. Analysis of Chocolate Hardness

A texture analyzer (TA.XT plus, Stable Micro Systems Ltd., Surrey, UK) was used to measure the hardness of the chocolates according to Chen et al. [21]. A P/2 cylindrical probe penetrated 4 mm into the sample at a rate of 2 mm/s. The peak force (g) was identified as hardness. In order to compare the hardness at different temperatures, chocolate samples were stored at 15 °C, 25 °C and 32 °C for 8 h and tested rapidly at room temperature (20 °C). Each analysis was repeated at least three times.

### 2.9. Determination of Differential Scanning Calorimetry (DSC)

A differential scanning calorimeter (DSC 4000, PerkinElmer, Waltham, MA, USA) was used to evaluate the melting properties of each chocolate. The chocolates were crushed in a pre-cooled mortar at 4 °C, mixed evenly and put in an aluminum crucible. The samples were stabilized at 20 °C for 5 min and then heated to 60 °C at a rate of 10 °C/min to obtain a melting curve [8,22]. Enthalpy and peak temperatures were calculated from the curve using Pyris series 11.0 software.

### 2.10. Statistics

The experiments were conducted at least twice, and all the data were reported as mean ± standard deviation (SD). SPSS 27.0 software was used for one-way ANOVA at a significance level of 5%.

## 3. Results and Discussion

### 3.1. Lipid Compositions of Mangosteen Seed Fat

StOSt and StLSt differ by only one double bond in sn-2 fatty acid. Takeuchi et al. [17] found that the mixture of the two has unique properties in terms of crystallization, which was attributed to oleic acid chain interactions with the linoleic acid chain. CB contained a large amount of SUS (S, saturated fatty acid; U, unsaturated fatty acid) triglycerides, which also suggests that StLSt might interact specifically with CB and affect the crystallization behaviors of the fat matrix.

As shown in Table 1, CB was mainly composed of palmitic acid (25.85%), stearic acid (36.63%) and oleic acid (32.61%), and the triglyceride results (Table 2) show that it possessed about 95.78% SUS-type triglycerides (POP, 18.36%; POSt, 48.25%; StOSt, 27.64%). This composition of CB was similar to that reported in the literature [23].

MSF contained both saturated fatty acids (stearic acid, 56.98%; and palmitic acid, 5.08%) and unsaturated fatty acids (oleic acid, 22.85%; and linoleic acid, 13.83%), which was similar to the fatty acid compositions of Thai MSF reported previously, but with slight variations in the content of stearic acid (47.6–57.9%), oleic acid (16.0–28.6%) and linoleic acid (14.7–20.3%) [14,15]. In contrast, some reported mangosteens native to Africa belong to *Garcinia Livingstonei*, and their fat exhibited different fatty acid compositions [24].

In the present study, the contents of StLSt and StOSt in MSF were the highest, accounting for 30.43% and 48.04%, respectively, followed by POSt (6.51%), POP (5.04%), StOO (4.00%) and PLSt (3.59%). Chen et al. [15] reported MSF with similar dominant triacylglycerols, mainly StLSt (31.67%), StOSt (31.47%), PLSt (12.31%), POSt (7.82%) and StOL (6.63%). This difference may be related to the cultivation conditions of mangosteens. The physicochemical properties and practical applications of tropical seed fats are strongly influenced by their fatty acid compositions [12]. With the addition of MSF, the fatty acid ratio of the binary blends showed a regular change, and it was also expected to explain the role of MSF in the crystallization of CB.

### 3.2. Solid Fat Contents of the Binary Fats

**Figure 1:**
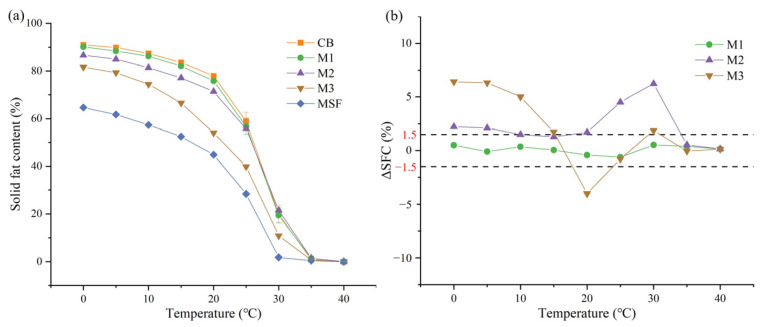
(**a**) Solid fat contents of mangosteen seed fat (MSF), cocoa butter (CB) and their binary blends; (**b**) ΔSFC values of the binary blends consisting of mangosteen seed fat and cocoa butter.

The binary fats showed similar melting curves to CB and MSF, but there were significant differences in the solid fat content values (Figure 1a). At 20 °C, the SFC values differed by more than 17.3% among the samples, which may cause a significant decrease in hardness. Based on the analysis of triglyceride compositions, this might be because there was a certain amount of StOO (4.00%) and PLSt (3.59%) in MSF, and they tended to retard the crystallization process or form lower melting point crystals [25,26].

It was worth noting that the fats containing 25% or less MSF had the most similar melting characteristics to CB, with a sharp drop in SFC values in a narrow temperature range of 25–35 °C, from 55.8 to 58.9% to 0.9–1.4%; the change in M3 was similar but with lower SFC values, indicating that its chocolate may be softer when it is first taken in the mouth. All the samples showed SFC values of nearly zero at 35 °C, proving that they presented good melting properties and will not cause a waxy feeling in the mouth. In general, the SFCs at each temperature decreased with increasing proportions of MSF in the binary blends, indicating that the two fats had the potential to be miscible at the studied blending ratios. Furthermore, when the ΔSFC value is in the low range, it indicates that the fat blends can achieve acceptable miscibility [20]. The ΔSFC values of binary fat blends were in the low range in this case (Figure 1b).

### 3.3. Crystal Morphologies of the Binary Fat Crystals

**Figure 2:**
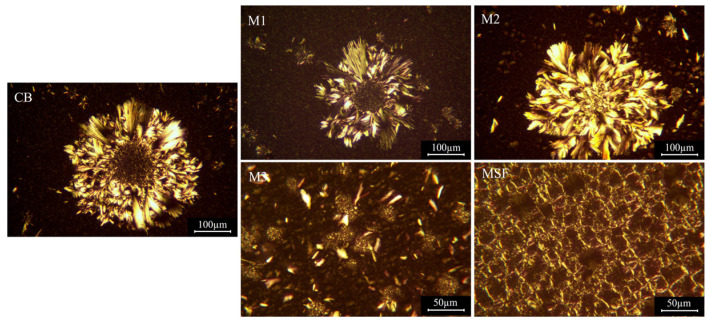
Crystal morphologies of mangosteen seed fat (MSF), cocoa butter (CB) and their binary blends.

After being stored at 20 °C for 14 days, as shown in Figure 2, MSF showed a fine needle-like crystalline structure, which may indicate the presence of γ crystal phase in the system [15,27]. MSF contained a relatively high content of linoleic acid and was prone to forming a more fine and dense structure [15]. In the binary blends, increased oleic acid levels changed crystal morphology significantly. CB, M1 and M2 shared similar large crystal morphologies (nearly 250 nm), characterized by granular centers with layers of feather-like structures as reported by Ramel et al. [28], which may belong to typical β crystal morphology.

In contrast, M3 contained smaller fat crystals (less than 50 nm), which was also present in M2. The morphologies were similar to that of cocoa butter when stored at 26 °C for 14 days [27], indicating that the system may have undergone a transformation from γ to β crystal form. The same crystals may also show different crystal morphologies due to different nucleation and growth modes [28,29].

### 3.4. Bloom Behaviors of Mangosteen Seed Fat-Based Chocolates

**Figure 3:**
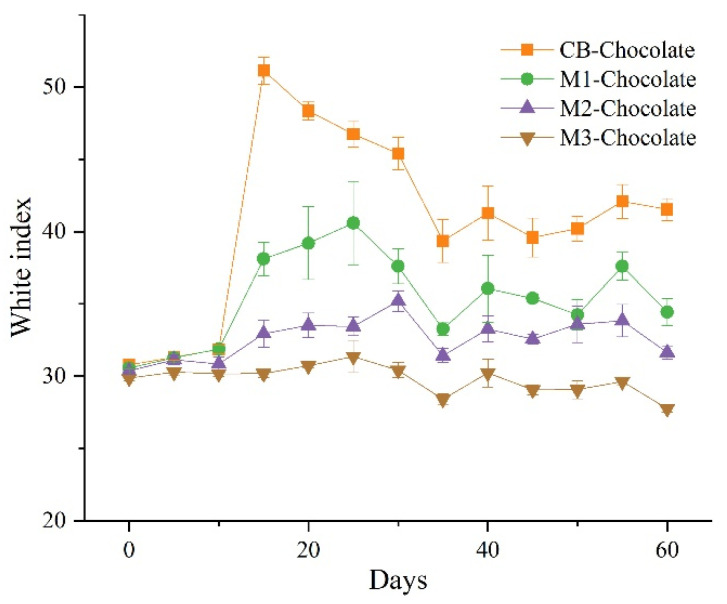
Whiteness indices of chocolates stored at cyclic temperatures.

**Figure 4:**
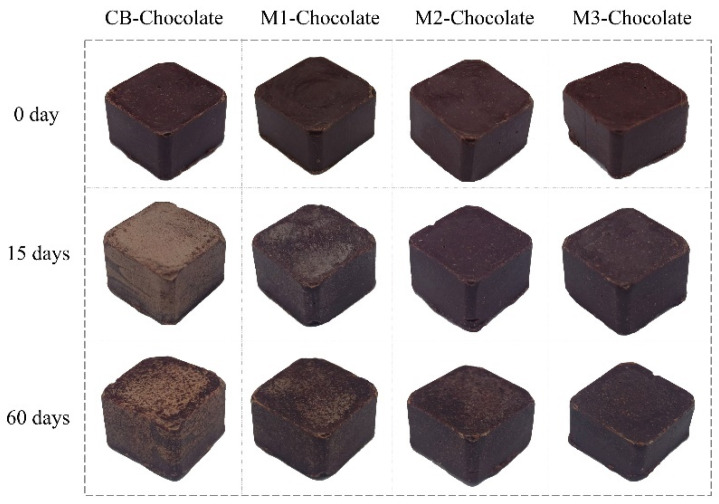
Surface appearance of chocolates stored at cyclic temperatures.

Figure 3 shows the whiteness index changes in the chocolates incorporated with MSF when stored at cyclic temperatures (17 °C–32 °C, 12 h). On the 15th day, the WI of CB-chocolate increased sharply to 51.13, while the WI change of M1-chocolate was about one-third of that of CB-chocolate. Throughout the storage process, the WI of M3-chocolate fluctuated little and there was no obvious bloom on its surface (Figure 4). In general, as the added amounts of MSF increased, the WIs of the related chocolates gradually decreased, indicating that MSF can delay the development of fat bloom.

Although the bloom degrees of CB-chocolate and M1-chocolate on day 60 were weaker than after 15-day storage, the surfaces of the chocolates were obviously rougher. Shen et al. [30] surmised that these changes in surface roughness resulted from temperature fluctuation, made high-melting CB components melt at higher temperatures and caused further re-crystallization at temperature reduction conditions.

Kinta and Hatta [31] classified fat bloom into three types based on their morphologies. One of the typical blooms was dark brown spots distributed in light brown parts [32], which was consistent with the bloom performance of CB-chocolate and M1-chocolate. The phenomenon was attributed to the growth of fat crystals around limited β nuclei. It could be inferred that after 32 °C storage, there were insufficient β crystals in CB-chocolate and M1-chocolate, and the recrystallization of other fat crystals during temperature fluctuation resulted in an unstable fat matrix. The mechanisms for inhibiting bloom in M2-chocolate and M3-chocolate need further investigation.

### 3.5. Texture Characteristics of Mangosteen Seed Fat-Chocolates

To evaluate the texture of chocolates made from CB and MSF, a texture analyzer was used to monitor changes in stress during puncture, and the maximum values were regarded as the hardness of chocolates [21,33]. Prior to the determination, the samples were stored at 15 °C, 25 °C and 32 °C for 8 h to stimulate fat crystal development [34,35]. Table 3 shows that there were significant differences in the hardness of each chocolate after storage at the specified temperatures. At 15 °C, the hardness of M1-chocolate, M2-chocolate and CB-chocolate were similar, indicating that these chocolates possessed similar fracture properties. In the 25 °C group, with an increase in MSF, the hardness of chocolates showed a slow declining trend, indicating that MSF may soften fat crystal networks, forming slightly lower melting point crystals, but it may not affect the acceptability of chocolates [21]. At 32 °C, the hardness of all the chocolates decreased distinctly, while the differences were not obvious as the remaining solid fats of each chocolate were quite low.

### 3.6. Thermal Behaviors of Mangosteen Seed Fat-Chocolates

Differential scanning calorimetry has been extensively employed to analyze the melting behaviors of chocolates. The obtained melting curves can clearly reflect solid fat contents and crystal transformation information. There are six crystal forms of cocoa butter, among which the most desirable one is the β₂ crystal form. The corresponding melting points vary according to the cocoa source and determination method [36]. Table 4 shows that there were two endothermic peaks in CB-chocolate, with peak temperatures of 35.36 °C and 29.91 °C, respectively, indicating the presence of other crystals besides the β₂ form. Other chocolates had only one endothermic peak with a peak temperature range of 33.5 °C–34.1 °C, suggesting that the systems are stable in a desirable crystal state [37].

In the accelerated bloom test, the samples were exposed to a high temperature of 32 °C for 12 h, which was only 1.53–3.36 °C lower than the peak temperatures. This indicates that some fat crystals may melt during the process. Thus, the degree of oil migration and the differences in nucleation, growth and transformation during recrystallization are the key factors influencing chocolate bloom [32].

## 4. Conclusions

Replacing CB with 25–60% MSF contributed to delaying the bloom in chocolates effectively. CB-chocolate had the most serious bloom development, characterized as dark brown spots distributed in light brown parts, which was attributed to fluctuated storage temperatures. The bloom could be restricted by incorporating MSF, the dominant triacylglycerols of which are StLSt and StOSt. Although the hardness of MSF-based chocolates was slightly reduced at 15 °C and 25 °C compared with its CB counterpart, there were no significant differences at 32 °C. DSC further revealed that the peak melting points of CB-chocolate and M1-chocolate were slightly higher than those of the M2- and M3-samples. Accordingly, the binary fat blends exhibited a steep melting curve between 25 °C and 35 °C, but with the addition of MSF, the SFC values gradually decreased, suggesting that the low-melting triacylglycerols in MSF may delay the crystallization process. It is speculated that the inhibition of fat bloom is due to the effective steric hindrance of StLSt crystals, which contribute to improving fat compatibilities and further delay liquid–oil migration and recrystallization. The detailed mechanism needs further research. This study provided a reference for the development of mangosteen seed fat, reduced the waste of resources to a certain extent and added information for research on chocolate fat bloom.

## Figures and Tables

**Table 1:** Fatty acid compositions of cocoa butter, mangosteen seed fat and their binary blends.

	CB	M1	M2	M3	MSF
P	25.85 ± 0.01	24.90 ± 0.05	20.94 ± 0.04	13.77 ± 0.05	5.08 ± 0.01
St	36.63 ± 0.06	37.52 ± 0.01	41.44 ± 0.01	48.47 ± 0.07	56.98 ± 0.08
O	32.61 ± 0.04	32.19 ± 0.01	30.31 ± 0.04	26.91 ± 0.01	22.85 ± 0.04
L	2.35 ± 0.01	2.89 ± 0.00	5.06 ± 0.01	9.04 ± 0.01	13.83 ± 0.07
Ln	1.29 ± 0.01	1.26 ± 0.01	1.16 ± 0.00	0.98 ± 0.01	0.75 ± 0.00
A	0.22 ± 0.00	0.22 ± 0.00	0.19 ± 0.01	0.12 ± 0.01	0.04 ± 0.01
Others	1.06 ± 0.03	1.03 ± 0.01	0.92 ± 0.01	0.73 ± 0.01	0.48 ± 0.01

CB, cocoa butter; MSF, mangosteen seed fat; P, palmitic; St, stearic; O, oleic; L, linoleic; Ln, linolenic; A, arachidic; *n* = 3.

**Table 2:** Triacylglycerol compositions of cocoa butter and mangosteen seed fat.

Triacylglycerol (%)	CB	MSF
PLP	0.29 ± 0.05	1.33 ± 0.06
POO	1.05 ± 0.01	0.37 ± 0.07
PLSt	1.24 ± 0.06	3.59 ± 0.19
POP	18.36 ± 0.16	5.04 ± 0.19
StOO	1.34 ± 0.00	4.00 ± 0.09
POSt	48.25 ± 0.49	6.51 ± 0.67
StLSt	—	30.43 ± 0.41
PPSt	0.66 ± 0.08	—
StOSt	27.64 ± 0.58	48.04 ± 0.19
StOA	0.54 ± 0.08	—
Others	0.66 ± 0.04	0.68 ± 0.06

CB, cocoa butter; MSF, mangosteen seed fat; P, palmitic; St, stearic; O, oleic; L, linoleic; A, arachidic; —, not analyzed; *n* = 3.

**Table 3:** Chocolate hardnesses when stored at 15 °C, 25 °C, 32 °C.

Hardness (g)	CB-Chocolate	M1-Chocolate	M2-Chocolate	M3-Chocolate
15 °C	5041.63 ± 765.63	4898.82 ± 575.11	5180.04 ± 504.82	4227.98 ± 1224.89
25 °C	2337.87 ± 126.92	1996.69 ± 105.30 *	1888.76 ± 176.70 *	1758.25 ± 87.73 *
32 °C	136.28 ± 15.73	163.12 ± 29.76	151.83 ± 26.81	124.88 ± 17.99

Values with * mean significant differences at 5% level between mixed samples and CB.

**Table 4:** Melting parameters of chocolates.

	Onset Temp. (°C)	Peak Temp. (°C)	Enthalpy (J/g)
CB-chocolate	33.05 ± 0.35	35.36 ± 0.11	25.01 ± 3.26
	25.28 ± 0.45	29.91 ± 0.10	15.57 ± 4.47
M1-chocolate	27.83 ± 0.25	34.07 ± 0.88	37.47 ± 1.07
M2-chocolate	29.73 ± 0.49	33.53 ± 0.11	38.83 ± 0.13
M3-chocolate	29.08 ± 0.42	33.95 ± 0.01	35.00 ± 1.67

## Data Availability

The original contributions presented in this study are included in the article. Further inquiries can be directed to the corresponding author.

## Appendix A

**Table A1:** Thermal and structural information of polymorphic forms of StOSt and StLSt [16,17].

		T_m_ (°C)	ΔH_m_ (kJ/mol)	LS (nm)	SS (nm)
StOSt	α	23.5	47.7	4.8	0.421
	γ	35.4	98.5	7.1	0.472 0.450 0.388
	β’	36.5	104.8	7.0	0.424 0.390
	β_2_	41.0	143.0	6.5	0.458 0.367
	β_1_	43.0	151.0	6.5	0.458 0.365
StLSt	sub-α₂	−5.3	3.7	—	0.419 0.374
	sub-α₁	8.8	4.2	—	0.421 0.385
	α	20.8	40.9	5.4	0.412
	γ	34.5	137.4	7.3	0.474 0.450 0.381

StOSt, 1,3-stearoyl-2-oleoyl-glycerol; StLSt, 1,3-distearoyl-sn-2-linoleoyl-glycerol (StLSt); T_m_, melting point; ∆H_m_, enthalpy of melting; LS, long-spacing value; SS, short-spacing value; —, no data.
